# Human Cytomegalovirus Induces Cellular and Humoral Virus-specific Immune Responses in Humanized BLT Mice

**DOI:** 10.1038/s41598-017-01051-5

**Published:** 2017-04-20

**Authors:** Lindsey B. Crawford, Rebecca Tempel, Daniel N. Streblow, Craig Kreklywich, Patricia Smith, Louis J. Picker, Jay A. Nelson, Patrizia Caposio

**Affiliations:** Vaccine and Gene Therapy Institute, Oregon Health & Science University, Beaverton, Oregon 97006 USA

## Abstract

The strict species specificity of Human Cytomegalovirus (HCMV) has impeded our understanding of antiviral adaptive immune responses in the context of a human immune system. We have previously shown that HCMV infection of human hematopoietic progenitor cells engrafted in immune deficient mice (huNSG) results in viral latency that can be reactivated following G-CSF treatment. In this study, we characterized the functional human adaptive immune responses in HCMV latently-infected huBLT (humanized Bone marrow-Liver-Thymus) mice. Following infection, huBLT mice generate human effector and central memory CD4+ and CD8+ T-cell responses reactive to peptides corresponding to both IE and pp65 proteins. Additionally, both HCMV specific IgM and IgG B-cell responses with the ability to neutralize virus were detected. These results indicate that the HCMV huBLT mouse model may provide a valuable tool to study viral latency and reactivation as well as evaluate HCMV vaccines and immune responses in the context of a functional human immune system.

## Introduction

Human cytomegalovirus (HCMV) is a prototypical betaherpesvirus and a ubiquitous opportunistic pathogen. Populations susceptible to severe HCMV infections include transplant recipients undergoing immunosuppressive therapy, HIV-infected individuals, and the developing fetus^1^. Specific immunological determinants that predispose individuals to infection and disease remain incompletely characterized. However, CD8+ and CD4+ T-cell responses, antiviral antibodies, and natural cytotoxicity have all been shown to have a potential role in controlling HCMV replication^2^. Following primary CMV infection, the virus establishes a large CD4+ and CD8+ T-cell response that is maintained for the life of the host^3^. In CMV infected individuals, both the CD4 and CD8 memory T-cell compartments including blood and tissues contain approximately 10% CMV-specific CD8 T-cells^4^. These anti-CMV T-cell responses are phenotypically unique, characterized by their mature effector memory phenotype. Interestingly, these responses expand over time thus overcoming normal T-cell exhaustion. Similarly, during maturation of the immune response in murine cytomegalovirus (MCMV)-infected mice, CMV-specific CD8+ T-cells assume a steadily increasing percentage of the overall T-cell pool in a process termed “memory inflation”^5^ (reviewed by ref. 6). The development of CMV-specific T-cell responses in rhesus macaques is slightly different as both CD4+ and CD8+ CMV-specific T-cells appear at high frequency during primary infection and then persist indefinitely at high levels^7^.

Generation of huBLT mice has been instrumental for the direct *in vivo* investigation of viruses with growth restricted to human cells. Development of humanized mouse models in which mice are engrafted with human cells or tissues have been shown to be capable of supporting human-tropic viral infections and modeling the human immune response for a number of viruses in the relevant cellular contexts^8–21^. The strict species specificity of HCMV and the lack of surrogate CMV animal models have driven the development of humanized mouse models in which mice are engrafted with human cells or tissues capable of supporting local HCMV infection (reviewed in ref. 22). The original HCMV humanized mouse models involved SCID (severe combined immunodeficient) mice engrafted with either human peripheral blood leukocytes (SCID-hu-PBL model) or with human fetal thymic and liver tissues (SCID-huThy/Liv model)^23–25^. Mocarski *et al*. utilized a SCID-huThy/Liv mouse model to assess the ability of the Toledo strain of HCMV to replicate within human fetal tissue implants^26^. In a separate study, Brown *et al*. utilized a SCID-huThy/Liv mouse model to evaluate and compare the replicative capacity of a low-passage Toledo strain of HCMV and high-passage, laboratory-adapted HCMV strains AD169 and Towne^27^. Recently, Dulal *et al*. reported a functional analysis of HCMV UL/b’ region using SCID-hu mice transplanted with human fetal thymus and livers tissues directly inoculated with purified virus^28^. These humanized mouse models had several limitations including lack of long-term human cell engraftment, low diversity in the types of cells engrafted, lack of distribution of human cells throughout the mouse and inability to generate human immune responses.

Over the past decade, humanized mouse models have been developed in which immune deficient mice have been engrafted with primary human hematopoietic progenitor cells (HPCs) with the goal of recapitulating a functional human immune system. Advancements relating to xenograft tolerance and xenograft tissue functions have allowed high levels of human chimerism, especially with respect to immune cells and liver tissue. The biggest breakthrough occurred with the development of an immune deficient mouse with a mutation in the interleukin-2 receptor *γ*-chain locus (*IL-2γc* ^*−*/*−*^). These mice exhibited a severe impairment of mouse B, T, and NK cell development allowing greater retention of HPC allografts in additional to a greater lifespan and reduced GVHD^29, 30^. Three main mouse strains have been developed with the *IL-2γc* ^*−*/*−*^ mutation including NOD.Cg-*Prkdc* ^*scid*^ *IL2rγ* ^*tm1Wjl*^ (NSG), NOD.Cg-*Prkdc* ^*scid*^ *IL2rγ* ^*tm1Sug*^ (NOG) and strains based on C;129S4-*Rag2* ^*tm1Flv*^ *IL2rγ* ^*tm1Flv*^ (RG). Each of these mouse strains exhibit differences in human immune system cell development. For example, NSG mice support higher levels of HSC engraftment and T-cell development in comparison to RG mice. NSG mice also have increased HSC bone marrow engraftment in comparison to NOG mice^29, 31^. Analysis of human hematopoietic cells demonstrated that these mice reconstituted monocytes, macrophages and B-cells as well as limited T-cells. The limit in T-cell maturation is believed to be due to education of these cells in the mouse thymus in the context of mouse MHC I and II. We previously reported the first humanized mouse model in which NSG mice engrafted with human CD34 + hematopoietic progenitor cells (HPCs) (huNSG) can be infected with HCMV and support a latent viral infection that can be reactivated in human macrophages following granulocyte-colony stimulating factor (G-CSF)-induced mobilization of HPCs^32^. While huNSG mice are useful to analyze HCMV infection, these mice are limited due to the lack of functional B-cells, CD4+ and CD8+ T-cells, dendritic cells, and limited reconstitution of endothelial and epithelial cells. Due to the lack of functional immune cells and the lack in supporting human cell types, huNSG mice are unable to develop complete T-cell responses and do not support antibody maturation. This limitation was overcome with the development of humanized mice that have been reconstituted with human fetal bone marrow, liver and thymus tissue (BLT)^33^. The huBLT mouse model represents a significant improvement over the huNSG model since huBLT mice exhibit improved systemic reconstitution of human hematopoietic cells including myeloid lineage cells, NK cells and CD4+ and CD8+ T-cells due, in part, to the presence of human thymic epithelium.

Multiple groups have utilized the huBLT model to assess the virological and immunological responses to HIV and provide convincing evidence that huBLT mice are a robust model to study human immune responses to a human-tropic pathogens including HIV^34^, EBV^15^, KSHV^16^, Dengue^17^ and Ebola^21^. Studies of herpesvirus infection in huBLT mice, however, are limited to two studies. Wang *et al*. demonstrated that huBLT mice can be infected with KSHV^16^ and, by microscopy, demonstrated that B-cells, macrophages and endothelial cells can support both latent and lytic infection. None of the mice in this study demonstrated a humoral immune response, or KSHV-related disease symptoms and cellular immune responses were not evaluated. In addition, Melkus *et al*. demonstrated that huBLT mice can be infected with EBV and generate virus specific MHC-restricted T-cell responses^15^.

In this study, we characterized the functional human adaptive immune responses in HCMV latently-infected huBLT mice. We observed that latently infected huBLT mice generate central and effector memory T-cells that are specific for HCMV as well as induce the production of HCMV-specific IgM and IgG neutralizing antibodies. These results indicate that the HCMV/huBLT mouse infection model provides a robust small animal model to analyze HCMV immune responses following infection and test candidate HCMV vaccines.

## Results

### Human cell reconstitution in huBLT mice

**Figure 1:**
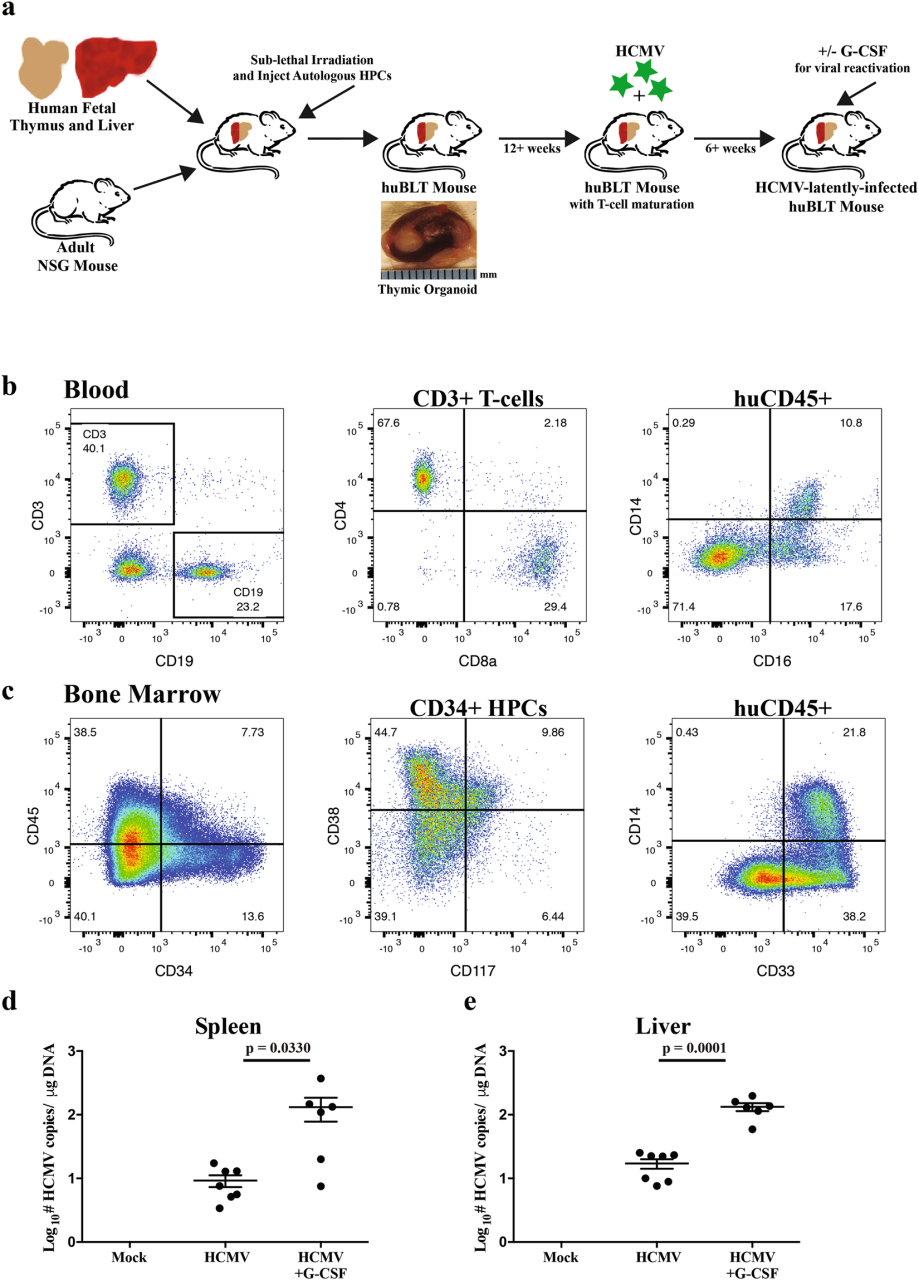
Generation of humanized BLT-NSG (huBLT) mice and human cell reconstitution. (**a**) Bone marrow-Liver-Thymus (huBLT) mice were generated by transplantation of human fetal liver and thymus under the kidney capsule of an adult NSG mouse. Post-surgical transplant, mice were sublethally irradiated (200 cGy) and intravenously injected with human CD34+ hematopoietic progenitor cells (HPCs) isolated from autologous fetal liver tissue. Post T-cell reconstitution (12–16 weeks following engraftment), huBLT mice were infected with HCMV by intraperitoneal (IP) injection of HCMV-infected fibroblasts or Mock infected by IP injection of uninfected fibroblasts. Beginning at 6 weeks post-infection huBLT mice are screened for HCMV-specific immune responses. Latently-infected huBLT mice are treated with G-CSF to induce viral reactivation at 8 weeks post-infection. (**b**) Human cell reconstitution was monitored by flow cytometry analysis of peripheral blood for the percentage of human CD45+ leukocytes (out of total human plus murine CD45+ leukocytes) beginning at 8 weeks post-humanization. Human CD45+ leukocytes can be further analyzed using human specific antibodies, including assessment of CD3+ T-cells and CD19+ B-cells (panel 1) and CD3+ T-cells further discriminated into CD4 and CD8 subsets (panel 2). In addition, monocyte subsets, as characterized by CD14 and CD16 staining, are present in the periphery (panel 3). **C)** Progenitor cell reconstitution was analyzed in the bone marrow using antibodies for human CD45 and CD34 (panel 1). CD34+ HPCs were further analyzed using antibodies for CD117 (c-kit) and CD38 (panel 2), and monocyte subsets cells analyzed using antibodies for CD33 (early) and CD14 (maturing) (panel 3). Data shown in (**b** and **c**) is from a huBLT mouse (cohort 3) at 17 weeks post-humanization gated on viable, muCD45- leukocytes. huBLT mice were divided equally into experimental groups based on overall human leukocyte reconstitution (human CD45+) and human T-cell reconstitution (human CD3+) in the periphery. At 8 weeks post-infection, huBLT mice (cohort 2) were reactivated by treatment with G-CSF and AMD3100. Seven days post-reactivation, all mice were euthanized and lymphoid organs collected. Genomic DNA was isolated using DNAzol and viral load determined by qPCR using primers and probe specific for HCMV UL144. Each sample was analyzed in triplicate. Data is shown for the average with standard error of the mean of two spleen tissue sections (**d**) or four liver tissue sections (**e**) per mouse (HCMV, n = 7; HCMV + G-CSF, n = 6), normalized to 1 ug of input DNA. Statistical analysis performed by one-way ANOVA.

The huBLT mouse represents a significant improvement over the huNSG model since the mice exhibit improved systemic reconstitution and functionality of human hematopoietic cells. To generate huBLT mice, adult NOD.Cg-*Prkdc* ^*scid*^ *IL2rγc* ^*tm1Wjl*^ (NSG) mice were transplanted with human fetal liver and thymic tissue under the right kidney capsule. Two weeks after transplant, the mice were sub-lethally irradiated and intravenously (IV) injected with autologous fetal liver-derived CD34+ HPCs (Fig. 1a). Human cell engraftment is visible in the periphery as early as 6 weeks post-transplant and functional T-cells are present in the periphery beginning around 12 weeks post-transplant. At this time, the human leukocyte ratio to total leukocytes is approximately than 50% in the peripheral blood. Four cohorts of huBLT mice were generated from four independent donor tissues with reconstitution of human T-cells, B-cells and monocytes evident in the periphery (Table 1). The average human cell reconstitution in the periphery at 12 weeks post-humanization for each cohort was (mean+/−SD) 23.2%+/−14.9% for cohort 1, 41.4%+/−21.3% for cohort 2, 54.2%+/−15.3% for cohort 3 and 63.5%+/−16.9% for cohort 4. huBLT mice developed a diverse repertoire of human lymphocytes including mature T and B-cells in the periphery (Fig. 1b, panel 1). By 12 weeks post-humanization, a significant proportion of the human lymphocytes in the periphery are T-cells (53.6%+/−29.4% for cohort 1, 50.7%+/− 24.3% for cohort 2, 37.1%+/−20.3% for cohort 3 and 37.4%+/−16.8% for cohort 4) and include both CD4 and CD8 single positive T-cells (Fig. 1b, panel 2). Mice with less than 10% human CD45+ lymphocytes or less than 10% human CD3+ T-cells at 12 weeks post-humanization were excluded from further analysis. Circulating classical and non-classical monocytes are also present in the periphery by 12 weeks (Fig. 1b, panel 3). Analysis of lymphoid organs (spleen, thymic organoid, lymph nodes) and solid organs (liver, lung) at necropsy demonstrate dispersal of human lymphocytes, monocytes and endothelial cells (data not shown and as previously described^15, 35^), while the bone marrow is repopulated with human stem and progenitor populations (Fig. 1c, panels 1 and 2) as well as lymphoid and myeloid precursors (Fig. 1c, panel 3).

**Table 1:** Human cell populations in huBLT mice cohorts.

huBLT Cohort	1		2		3		4	
	%	SD	%	SD	%	SD	%	SD
huCD45	23.3	14.9	41.4	21.3	54.2	15.3	63.5	16.9
CD3 + (gated on huCD45 + lymphocytes)	53.6	29.4	50.7	24.3	37.1	20.3	37.4	16.8
CD19 + (gated on huCD45 + lymphocytes)	40.0	26.7	44.0	24.3	54.7	17.5	55.2	19.9
N	22		35		29		11	

### HCMV Infection and T-cell response in huBLT mice

We have previously shown that HCMV can infect huNSG mice and upon mobilization of progenitor cells with G-CSF and AMD3100 the virus can be reactivated in the different humanized mouse tissues^32, 36, 37^. In order to assess the utility of the huBLT model to support HCMV infection and analyze antiviral immunity in this more immune-robust model, we infected huBLT mice with HCMV by intraperitoneal injection (IP) of HCMV-infected human fibroblasts. At 8 weeks post infection, huBLT mice were reactivated by treatment with G-CSF and AMD3100. Latency in the humanized mouse model is defined as the presence of viral DNA in bone marrow and spleen, the absence of viral mRNA (other than latency-specific transcripts), and the absence of viral dissemination to other tissues. In this model, HCMV reactivation is defined as the presence of viral DNA, mRNA and protein in tissues and macrophage-associated dissemination to other organs. Using this system, we are able to detect a quantitative increase in HCMV viral DNA load after reactivation in both the spleen (Fig. 1d; p = 0.0330) and liver (Fig. 1e; p = 0.0001) and mRNA expression (data not shown) following viral reactivation as compared to latently-infected huBLT mice. The detection of early and late HCMV transcripts in G-CSF treated mice demonstrates that the virus established latency in hematopoietic cells, which upon mobilization became permissive for HCMV replication and contribute to viral spread in the host.

**Figure 2:**
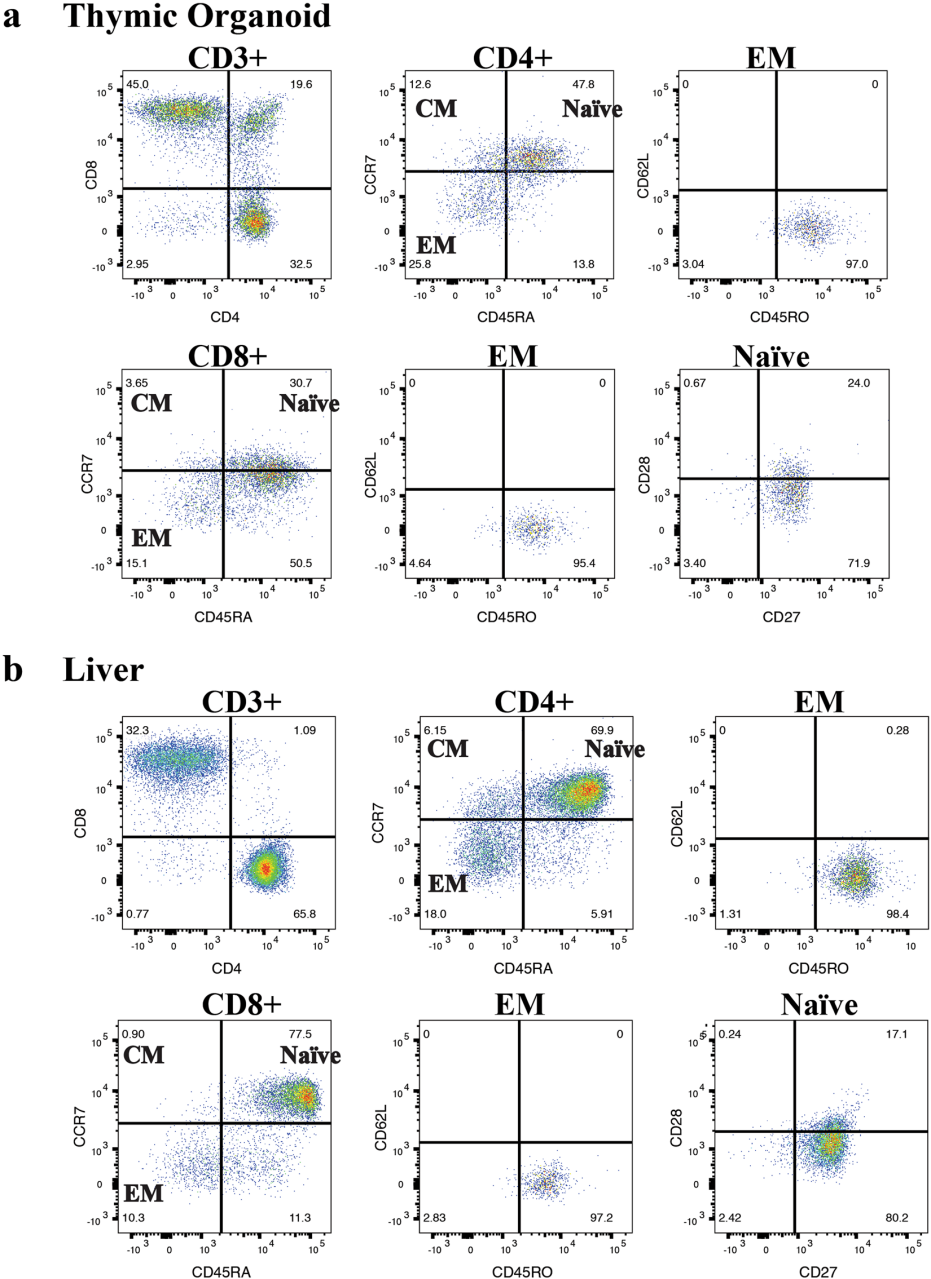
huBLT mice develop naïve, central memory and effector memory T-cells. huBLT mice were generated as described in Fig. 1. At 17 weeks post-humanization, total mononuclear cells from the thymic organoid (**a**) and liver (**b**) of an uninfected huBLT mouse (cohort 3) were analyzed by flow cytometry for human T-cell subsets. All samples were gated on total, viable, murine CD45−, human CD45+ leukocytes. Panel 1 shows CD4+ and CD8+ T-cell subsets (gated on CD3+ T-cells). CD4+ T-cell subsets (panels 2 and 3) can be further analyzed to assess T-cell development by staining for naïve (T_naïve_) (CD45RA + CCR7+), central memory (T_CM_) (CD45RA-CCR7+) and effector memory (T_EM_) (CD45RA-CCR7−) T-cells. Effector memory T-cells can be further classified by expression of CD45RO (panel 3). CD8+ T-cell subsets (panels 4 and 5) can be analyzed for naïve, central memory and effector memory T-cells as described above. Naïve CD8+ T-cells (CD45RA + CCR7+) can also be further classified by the expression of CD28 and CD27 (panel 6).

A major advantage of the huBLT model over other humanized mouse models is that human T-cells develop in an autologous human thymic environment. Analysis of the thymic organoid and splenic tissues in huBLT mice shows long-term T-cell maturation, development and reconstitution confirming previous studies demonstrating long-term thymopoiesis and development of diverse TCR T-cell repertoire^15^ and development of CD4 and CD8 specific immunity^12, 14, 15^. In our system, huBLT mice develop and demonstrate classical T-cell development (CD4/CD8 DN to DP to SP populations) in the thymic organoid (Fig. 2a, panel 1) with more mature SP populations in the periphery (Fig. 2b, panel 1). huBLT mice also maintain naïve, central memory and effector memory T-cells including both CD4+ T-cell populations in the thymic organoid (Fig. 2a, panels 2 and 3) and periphery (Fig. 2b, panels 2 and 3) and CD8+ T-cell populations in both the thymic organoid (Fig. 2a, panels 4 to 6) and periphery (Fig. 2b, panels 4 to 6).

**Figure 3:**
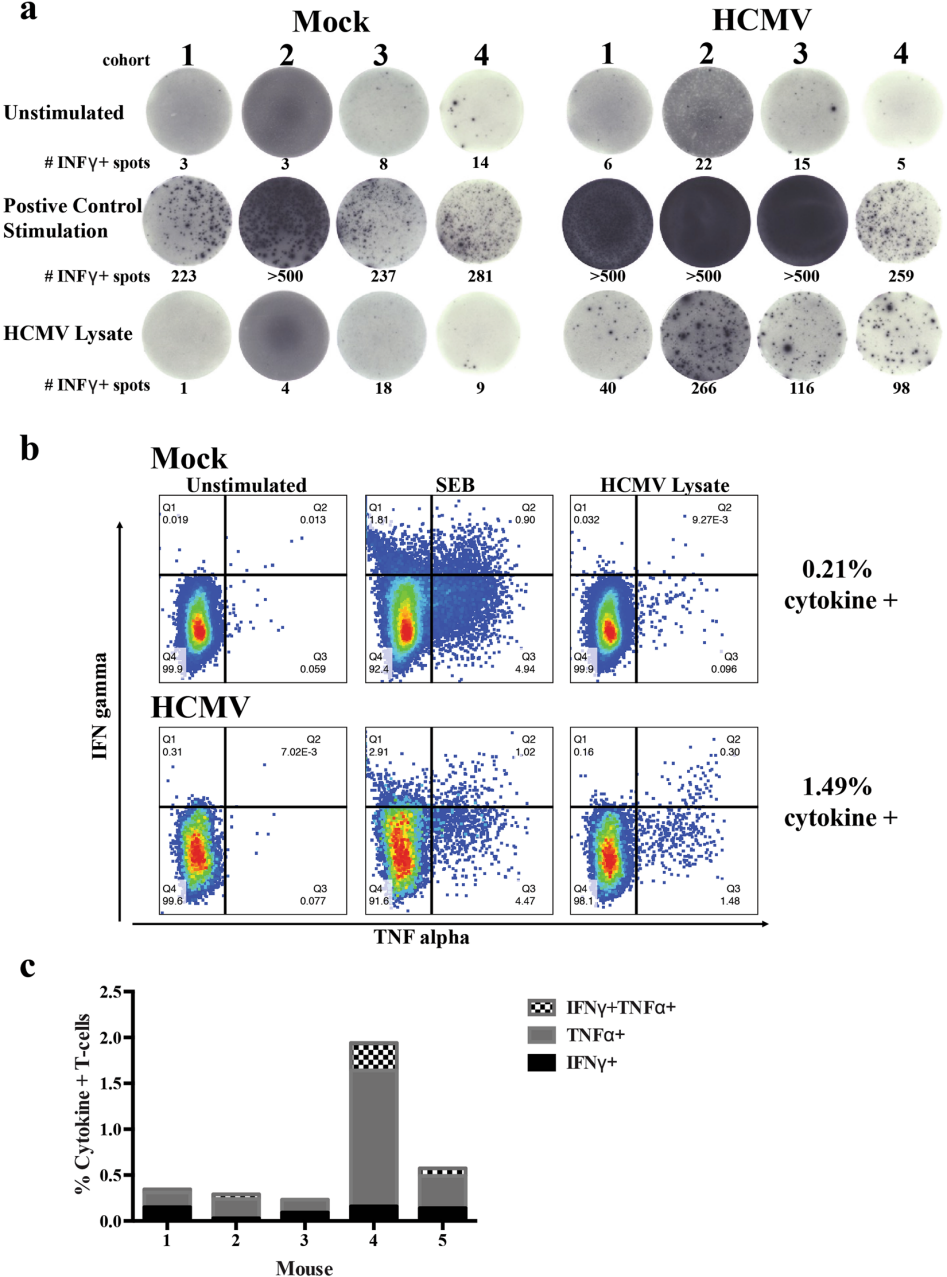
huBLT mice constructed from multiple donor tissues generate HCMV-specific multi-functional T-cell responses. (**a**) huBLT mice were generated and infected with HCMV as described in Fig. 1. Mice were euthanized at 8 weeks post-infection and splenocytes harvested. Total splenic mononuclear cells left unstimulated or were stimulated with SEB toxin or HCMV viral lysate and cultured on human IFNγ ELISpot plates (MabTech) prior to detection. Mice shown are from four independent experiments using separate donor tissues from Mock-infected (Mock, panel 1) and HCMV-infected (HCMV, panel 2) huBLT mice for each donor. (**b**) Mock-infected (top) or HCMV-infected (bottom) huBLT mice (cohort 3) were harvested at 6 weeks post-infection and splenocytes harvested. Total splenic mononuclear were unstimulated (panel 1) or stimulated with SEB toxin (panel 2) or HCMV viral lysate (panel 3), incubated with Brefeldin A and stained with human-specific antibodies for flow cytometry analysis of T-cell populations and cytokine production. Samples were gated on viable, human CD45+, CD3+ and CD69+ lymphocytes. (**c**) Quantification of the cytokine (IFNγ+, TNFα+ or IFNγ + TNFα+) responsive T-cells from five independent huBLT mice analyzed as in (**b**).

In order to determine whether HCMV-infected huBLT mice develop memory T-cells and generate specific anti-viral T-cell responses, we examined splenocytes from huBLT mice to determine if they respond to HCMV. Splenocytes were stimulated with either total viral lysate or specific viral peptides and assessed using enzyme-linked immune-spot (ELISPOT) analysis for production of human interferon γ (IFNγ). Lymphocytes isolated from the spleens of both uninfected (Fig. 3a, panel 1) and HCMV-infected (Fig. 3a, panel 2) huBLT mice produced IFNγ in response to SEB toxin stimulation. However, only splenocytes from HCMV-infected huBLT mice (Fig. 3a, panel 2) produced IFNγ in response to stimulation with HCMV lysate, indicating the generation of HCMV-specific T-cells following infection. HCMV-specific responses are detectable in four independent cohorts of HCMV-infected huBLT mice which confirms induction of T-cell responses to HCMV infection regardless of donor tissue background (Fig. 3a). Analysis of multi-functional T-cell responses by intracellular staining for both human IFNγ and human TNFα (Fig. 3b) indicates that HCMV-infected huBLT mice generate poly-functional T-cells in response to HCMV infection. Multiple individual huBLT mice are capable of generating multi-functional T-cell responses to HCMV (Fig. 3c).

**Figure 4:**
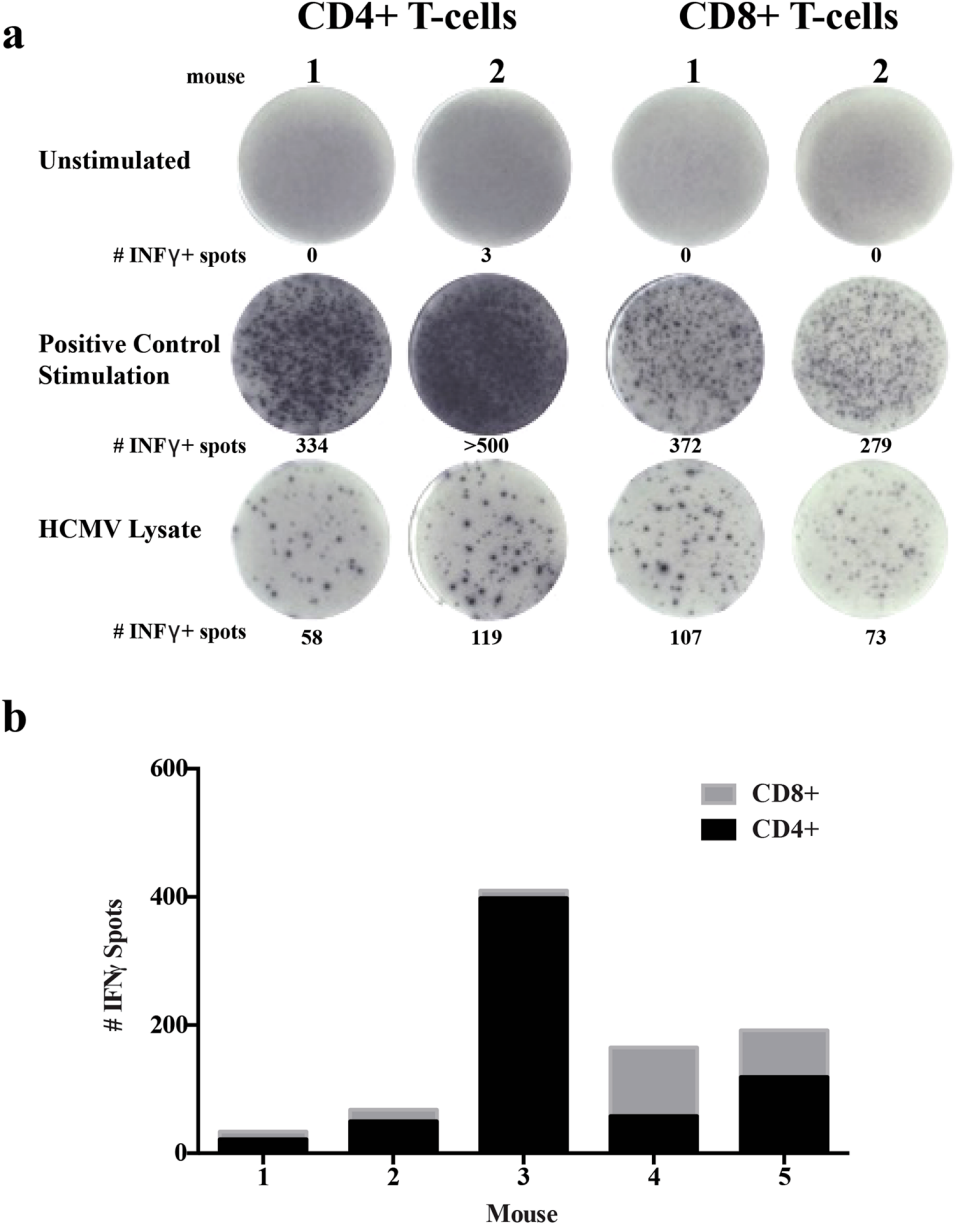
huBLT generate HCMV-specific human INFγ CD4+ and CD8+ T-cell responses. (**a**) huBLT mice were euthanized at 6 weeks post-infection and splenocytes harvested. Total splenic mononuclear cells were isolated using Ficoll and human CD4+ cells separated using magnetic bead isolation (Miltenyi Biotech) (panel 1). Human CD8+ cells (panel 2) were further isolated from the CD4- fraction also using magnetic bead isolation (Miltenyi Biotech) and cultured without stimulation (unstimulated), with positive control stimulation (SEB) or with HCMV lysate on human IFNγ ELISpot plates (MabTech) prior to detection, two independent mice are shown. (**b**) Quantification of the number of IFNγ positive spots produced by the CD4+ and CD8+ fractions from five independent mice.

In healthy humans, the CMV-specific T-cell response composes approximately 10% of both the CD4 and CD8 memory compartments to numerous CMV-encoded open reading frames^4^. To determine the specificity of the HCMV-specific T-cell responses in huBLT mice, we isolated CD4 and CD8 T-cell subsets from HCMV-infected huBLT mouse splenocytes. T-cell subsets were separated using magnetic bead antibodies by positive selection of CD4+ T-cells (CD4+ CD8− SP and CD4+CD8+ DP) followed by CD8 selection on the CD4 negative fraction (CD4-CD8+ SP T-cells). CD4+ and CD8+ T-cells were characterized for HCMV responses to viral lysate by ELISPOT analysis for human IFNγ. Representative images for two independent huBLT mice are shown in Fig. 4a and quantified for five independent huBLT mice in Fig. 4b. This data indicates that regardless of the degree of response, both CD4 and CD8 T-cell subsets generate HCMV-specific responses. The magnitude of the responses for the CD4 population is greater than for the CD8 T-cell population because whole HCMV lysate is poorly stimulatory for MHC class I-mediated responses^38^.

**Figure 5:**
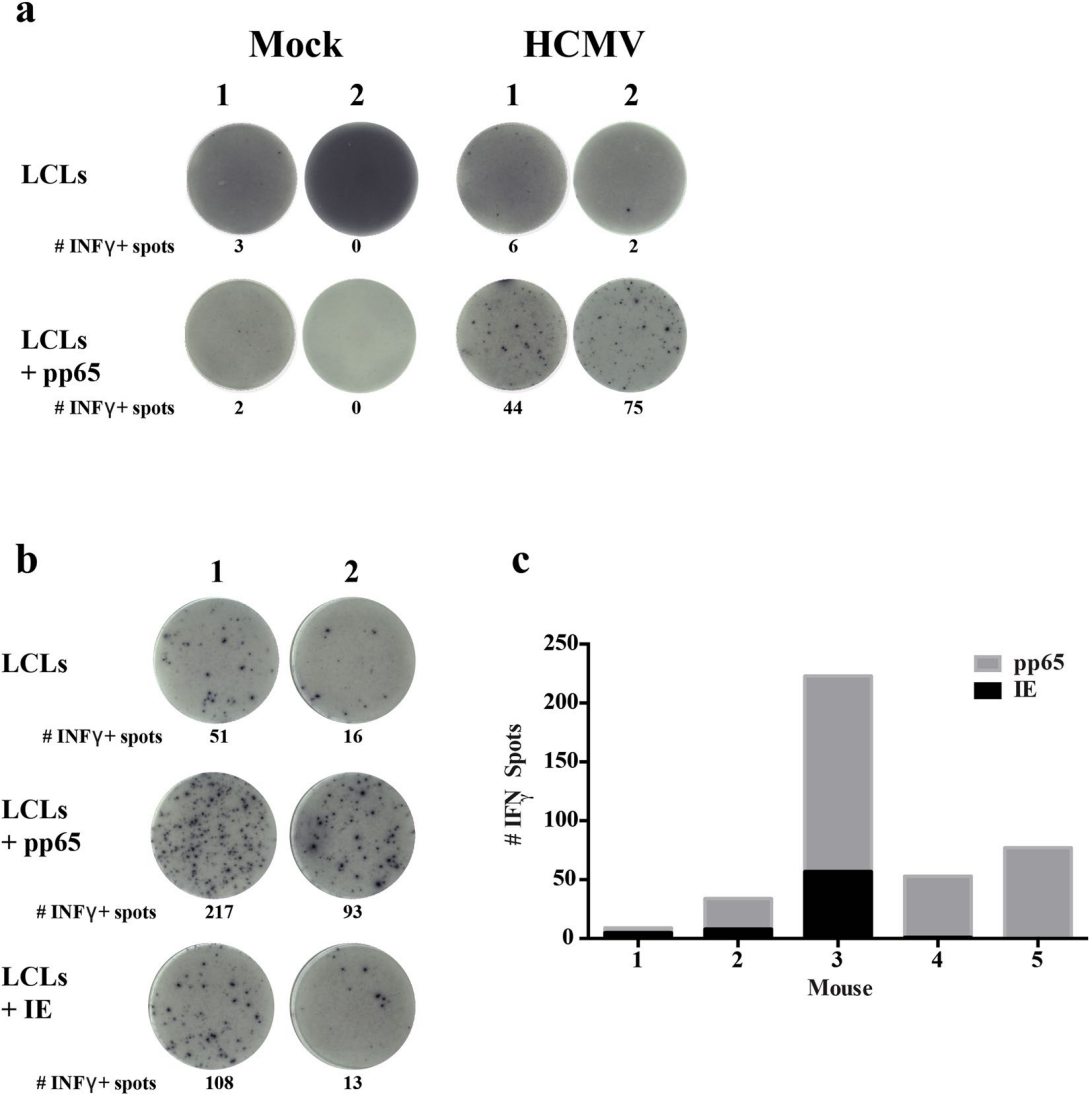
BLT Mice Generate Human IFNγ T-cell Responses Specific to HCMV pp65 and IE. (**a**) huBLT mice were generated and infected with HCMV as in Fig. 1, harvested at 6–8 weeks post-infection and splenic mononuclear cells isolated using Ficoll. Isolated splenocytes from two independent cohorts of huBLT mice were cultured with autologous LCLs alone or LCLs pre-loaded with HCMV pp65 peptides and cultured on human IFNγ ELISpot plates (MabTech) prior to detection. To determine the diversity of the HCMV-specific response, additional mice from one cohort of huBLT mice were analyzed for human IFNγ production in response to stimulation with LCLs presenting HCMV pp65 or IE. Two mice are shown in (**b**) and five mice are quantified in (**c**).

The breadth and complexity of HCMV responses in humans is diverse and involves a significant fraction of the viral protein repertoire with a variety of response intensities^39^. To determine the HCMV-specific T-cell response in huBLT mice, splenocytes from HCMV and Mock-infected huBLT mice were cultured with autologous LCLs pre-incubated with HCMV peptides (IE or pp65) prior to analysis by human IFNγ ELISPOT. In contrast to Mock-infected huBLT mice, HCMV-infected animals generated from multiple donor tissues generate specific responses to the late HCMV protein, pp65 (Fig. 5a). In addition, further analysis of HCMV-responding huBLT mice demonstrate specific responses to both immediate-early (IE) and late (pp65) HCMV antigen expression (Fig. 5b) with a variety of dominant responses between individual huBLT mice (Fig. 5c).

### Humoral Immune Response to HCMV

**Figure 6:**
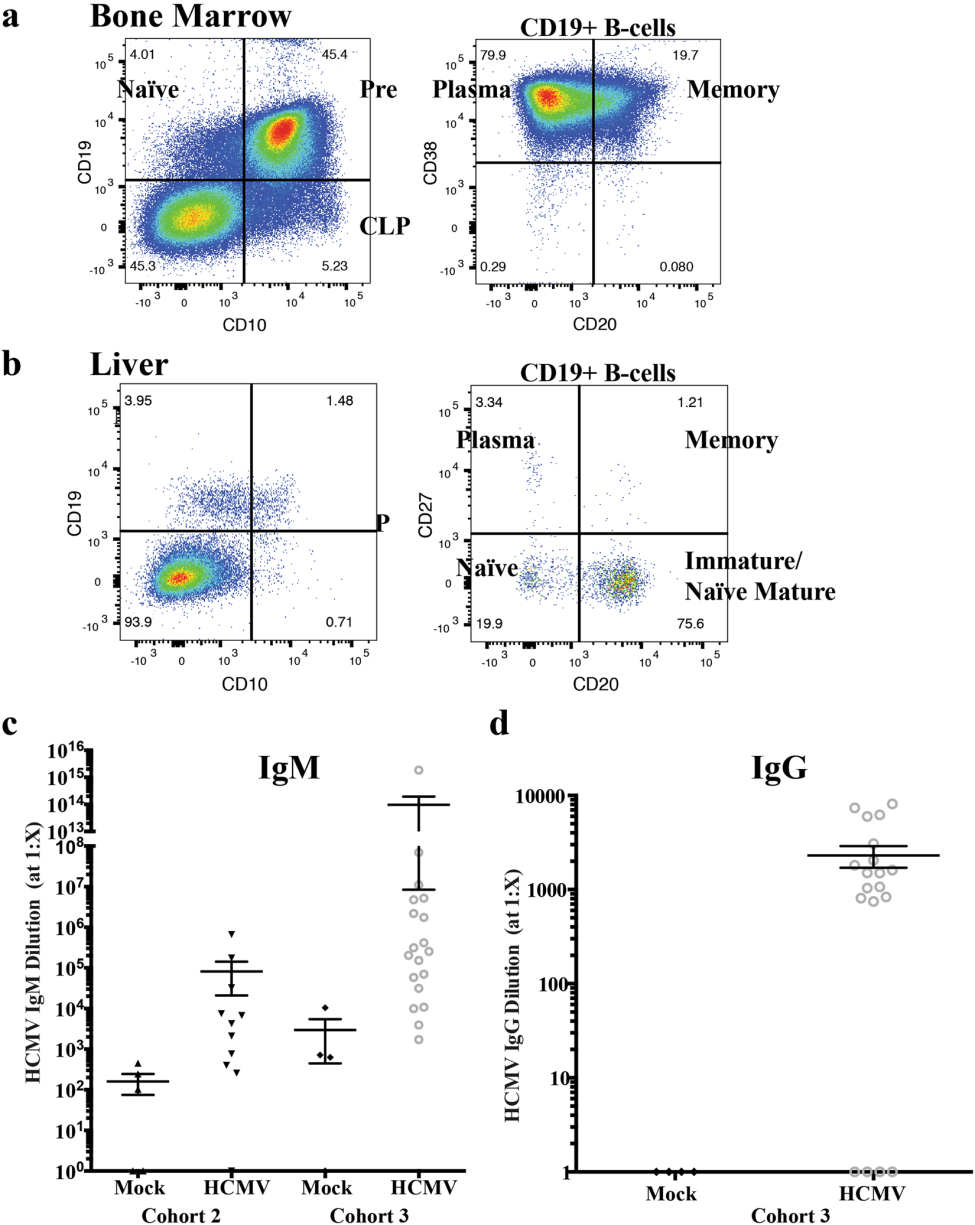
huBLT mice develop mature human B-cells and functional HCMV-specific antibody responses. huBLT mice were generated as described in Fig. 1. Total mononuclear cells from the bone marrow (**a**) and liver (**b**) from an uninfected huBLT mouse at 17 weeks post-humanization were analyzed by flow cytometry for human B-cell subsets. Samples were gated on total viable, murine CD45-, human CD45+ lymphocytes. Both organs were analyzed for B-cell maturation looking at CD10+ CD19- committed lymphoid progenitors (CLP, which are also CD38+ CD20−) and CD10+ CD19+ pre-B-cells and CD10-CD19+ naïve B-cells (panel 1). The CD19+ B-cell population was further analyzed for CD27-CD20- naïve B-cells, CD20 + CD27− immature/naïve mature B-cells, CD27 + CD20+ memory B-cells and CD27 + CD20− plasma B-cells (panel 2). huBLT mice were generated and infected with HCMV as described in Fig. 1 and euthanized at 8 weeks post-infection. Plasma samples were analyzed by ELISA for HCMV antibodies using a pan-IgG/IgM/IgA secondary antibody. Positive samples were re-analyzed for antibody maturation using secondary antibodies specific for IgM (**c**) or IgG (**d**).

Although huBLT mice are biased to T-cell lineage development due to the presence of robust thymic reconstitution and reduced lymph node size and development due to genetic mouse immunosuppression; lymph nodes are clearly reconstituted with human hematopoietic cells and some germinal center formation is observed^15^. B-cell production and maturation, however are a critical component to complete immune system responses. Importantly, during HCMV infection in humans, B-cell activation and subsequent antibody production are critical contributors to antiviral immunity and a potential target goal for vaccine development. Therefore, to investigate the development of mature human B-cells in our model, uninfected huBLT mice were analyzed by flow cytometry for human B-cell subsets. Flow cytometry analysis demonstrates that the bone marrow of huBLT mice contains multiple hematopoietic progenitor cell subsets (Fig. 1c) as well as naïve, pre-B and plasma B-cells (Fig. 6a). In addition, peripheral lymphoid tissues contain limited numbers of naïve and pre-B-cells but a significant proportion of immature and naïve, mature B-cells as well as plasma and germinal center B-cells (Fig. 6b).

In order to assess whether HCMV infection induces functional B-cell responses in huBLT mice, we characterized the antiviral antibody responses by ELISA and neutralization assays. Plasma from HCMV-infected or Mock-infected huBLT mice was analyzed for HCMV-specific antibodies using a pan-IgG/IgM/IgA secondary antibody. Positive samples were re-analyzed using specific IgM (Fig. 6c showing two independent cohorts of huBLT mice) and IgG (Fig. 6d) secondary antibodies. Importantly, analysis of huBLT mice infected with HCMV demonstrated that they generate HCMV-specific antibodies in the peripheral blood. Antibody isotype analysis revealed that the responses are both early (IgM) and later (IgG) isotype specific indicating maturation of the antibody response. Utilizing the HCMV envelope (gB) protein as the capture antigen for ELISA, we found that 50% of the animals had antibodies that were specific for HCMV gB (Table 2). Interestingly, more than half the animals developed neutralizing antibodies against HCMV (Table 2) similar to the response in HCMV infected humans^40^.

**Table 2:** HCMV-infected huBLT mice generate HCMV-specific antibody responses to HCMV GB and can neutralize live virus.

Mouse		IgM		IgG		GB		Neutralization	
Mock	1	7.17E + 02		0		NA		0	
Mock	2	1.05E + 04		0		NA		0	
HCMV	1	1.84E + 15	**	7384	**	0		NA	
HCMV	2	7.01E + 04		808	*	NA		1:128	**
HCMV	3	5.18E + 06	*	2055	*	NA		1:128	**
HCMV	4	1.10E + 07	*	1504	*	0		0	
HCMV	5	6.92E + 07	*	8144	**	7.35E + 01	*	0	
HCMV	6	3.13E + 04		1813	*	4.08E + 01	*	1:128	**
HCMV	7	5.83E + 04		5974	**	0		0	
HCMV	8	1.52E + 05	*	3079	**	0		1:32	*
HCMV	9	4.75E + 06	*	1037	*	4.06E + 02	**	1:16	*
HCMV	10	2.04E + 05	*	834	*	6.46E + 02	**	0	
HCMV	11	2.55E + 05	*	1496	*	5.74E + 01	*	1:16	*
HCMV	12	3.13E + 05	*	6205	**	0		1:64	**
HCMV	13	1.74E + 06	*	1607	*	0		1:16	*
HCMV	14	2.18E + 06	*	1068	*	3.32E + 02	**	0	

NA = not analyzed.

Negative.

*Positive, 50th percentile.

**Positive, High.

## Discussion

In summary we have shown that HCMV establishes latent infection of huBLT mice resulting in the generation of both human CD4+ and CD8+ T-cell responses as well as HCMV neutralizing IgM and IgG neutralizing antibodies. The establishment of the HCMV huBLT model is a significant advance in the field compared to previous models in which only immature T and B-cell developed due to the lack of a thymus^26, 27^. The HCMV huBLT model provides the first animal model to study human T and B-cell responses to HCMV infection as well as the development of new viral vaccines. In addition, the HCMV huBLT model provides a novel animal model to understand mechanisms of viral latency and reactivation in the context of an intact human immune system.

The huBLT mouse model has provided an important tool to examine virological and immunological aspects of human viruses that lack an animal model. The vast majority of the virus studies in huBLT mice have been with HIV. These studies include examining mechanisms of HIV pathogenesis and latency, viral mucosal transmission, and antiviral treatment^8–14^. HIV infected huBLT mice also the generated specific CD4+ and CD8+ cellular immune responses to multiple viral proteins including Gag, Pol, Env^8, 12, 14, 41^. The HIV-specific T-cell responses in huBLT mice were also functional as measured by their ability to exert strong selection pressure on acute HIV infection and by measurement of subsequent viral evolution and mutational escape similar to that seen in humans^14^. The huBLT model has also been used to examine infection with herpesviruses. Kaposi’s Sarcoma Herpesvirus (KSHV) infection of huBLT mice exhibited persistent infection of B-cells, macrophages and endothelial cells following mucosal infection^16^. EBV infection of huBLT mice resulted in the generation of human major histocompatibility complex class I and class II restricted T-cell responses to EBV^15^. However, this study did not characterize the EBV antigens that induce the response. In this study we show that huBLT mice are an ideal model to study HCMV latency resulting in a diverse immune response to HCMV with polyfunctional T-cells recognizing IE and the Early Late virion structural protein pp65 that are immunodominant proteins recognized by T-cells in asymptomatic humans (Fig. 5)^4^.

Antibody maturation in huBLT mice is somewhat controversial and may be dependent on the virus group and duration of infection. Humanized mice display normal B-cell maturation through the early stages of development in the bone marrow including B-cell receptor (BCR) diversity (reviewed by ref. 42). The generation of class-switched antibodies (IgM to IgG) occurs in huBLT mice that correlates with B-cell maturation status and time post-engraftment and develop functional, albeit rudimentary, germinal centers^8, 15, 32, 33, 40, 41^. Infection of huBLT mice with several different viruses has resulted in the induction of IgM but limited or undetectable IgG^17, 18, 20, 43^. In HIV infected huBLT mice IgM but not IgG antibodies increased significantly between 6 and 18 weeks post-infection^8^. Additional studies demonstrated that Dengue virus infection in huBLT mice induces broadly cross-reactive human IgM antibodies that recognized intact virions^17^. As shown in results HCMV latently infected mice develop a humoral immune responses composed of both IgM and IgG antibodies by 20 weeks post infection that neutralize HCMV gB one of the virion receptors necessary for viral entry into cells^2^. The ability of HCMV but not HIV to develop IgG neutralizing antibodies may involve the interval of time for antibodies to develop. Alternatively, HCMV IgG antibodies may develop because greater numbers of CD4+ T-cells necessary B-cell development in contrast to HIV infection of huBLT mice in which CD4+ T-cells are decreased due to the fact these cells are a target of HIV.

All CMVs express multiple viral proteins that inhibit antigen presentation by modulating major histocompatibility class I (MHC I) surface levels^44^. However, the role of these immune modulatory genes on T-cell responses and how they affect initial CMV infections, latency and reactivation are still unclear. Initially CMV immune evasion genes were hypothesized to be required for maintenance of a persistent CMV infection. However, studies with MCMV and RhCMV demonstrated that recombinant CMV lacking genes regulating MHC I expression were still capable of infecting CMV-naïve animals to establish persistent infections^45^. While the role of MHC I CD8+ T-cell responses against CMV is clearly evident, there is increasing evidence that CD4+ T-cells are also integral to the control of CMV infection and prevent against CMV disease^46^. In mice infected with MCMV, the selective depletion of CD4+ T-cells resulted in an increased incidence of recurrent MCMV infection^46^. CD4+ T-cells have also been shown to contribute to the control of primary MCMV infection in mice that had previously been depleted of CD8+ T-cells before infection^46, 47^. Although substantial progress has been made in characterizing the unique T-cell immunology of MCMV and RhCMV as well as the role of the MHC I inhibitory genes, analysis of HCMV has been limited because of the lack of an animal model. The HCMV huBLT model may provide a model to examine the immune response in the context of the MHC I inhibitory genes.

In summary the huBLT model provides the first animal model to explore the adaptive human immune response to HCMV infection. The ability to generate humanized mice will allow us to test different types of vaccines and formulations with important controls in the context of identical immune systems. The development of the huBLT model will also be important to test the immunogenicity of HCMV based vectors that express antigens of other pathogens or cancer antigens.

## Materials and Methods

### Ethics Statement

All animal experiments were conducted under the approved Oregon Health and Science University (OHSU) Institutional Animal Care and Use Committee protocol (3498). All mice in this study were managed in accordance with the NIH Office of Laboratory Animal Welfare: “PHS Policy on the Humane Care and Use of Research Animals” and the recommendations of the American Association for Accreditation of Laboratory Animal Care (AAALAC): “The Guide for the Care and Use of Laboratory Animals, 8^th^ edition”.

### Mice

NOD-*scid*IL2Rγc null (NSG) mice were purchased from Jackson Laboratories and bred in-house. All mice were housed in micro-isolator cages in a designated specific pathogen-free facility at OHSU and fed sterile food and water *ad litem*. Mice were euthanized via CO_2_ administration according to AAALAC euthanasia guidelines.

### Human Tissue Implants and CD34+ HPC isolation

Male and female NSG mice (5 to 23 weeks old) were anesthetized and ~1 mm pieces of human fetal thymus and liver tissue (17–24 weeks gestation, Advanced Bioscience Resources) were surgically implanted under the kidney capsule, essentially as previously described^32^. CD34+ hematopoietic progenitor cells (HPCs) were isolated from autologous fetal liver using human CD34 magnetic beads (Miltenyi Biotech) as previously described^48, 49^ and frozen at −80 °C until transplantation. Two weeks after surgical transplantation of tissues, mice were sublethally irradiated (200 cGy using a ^137^Cs gamma radiation source). At 24 hrs post-irradiation, mice were transplanted with ~10^5^ CD34+ HPCs via intravenous injection. Mice were screened for human cell engraftment beginning at 8 weeks post-transplantation as described below.

### Flow cytometry analysis of human cell reconstitution

Human cell reconstitution was assessed by flow cytometry on freshly isolated mononuclear cells obtained from blood, bone marrow, spleen and thymic organoid samples from huBLT or control mice. Non-specific antibody binding was blocked prior to and during staining using human and mouse serum. Flow cytometry analysis was performed using antibodies specific for the human cell surface markers CD3 (clone UCHT1), CD4 (OKT4), CD8 (HIT8a), CD10 (HI10a), CD14 (M5E2 or HCD14), CD16 (3G8), CD19 (HIB19), CD20 (2H7), CD27 (M-T271), CD29 (TS2/16), CD31 (WM59), CD33 (WM53), CD34 (581), CD38 (HIT2), CD45 (HI30), CD45RA (HI100), CD45RO (UCHL1), CD56 (HCD56), CD90 (5E10), and CD117 (104D2) (all from Biolegend); human cell intra-cellular markers CD69 (FN50, Biolegend), INFγ (B27, Becton Dickson), and TNFα (Mab11, eBioscience); and murine cell surface marker CD45 (Biolegend). Intracellular staining was performed after fixing and permeabilization using the BD Perm Fix kit (Becton Dickson). Viability was determined by staining with ZombieAqua viability dye (Biolegend) prior to antibody staining. All samples were fixed with 2% neutral buffered formalin prior to analysis. All data were collected and analyzed using an LSRII flow cytometer equipped with FACS Diva (Becton Dickson) and FlowJo v10 (TreeStar). Gates for human cell populations were set using equivalent stained samples from a non-humanized control NSG mouse and gates for murine cell populations were set using equivalent stained samples using human lymphocytes.

### Infection of huBLT mice with HCMV

Neonatal normal human dermal fibroblasts (NHDF) were cultured in DMEM (Cellgro) containing 10% FBS (Hyclone) and 1% penicillin, 1% streptomycin and 1% glutamine (Life Technologies) at 37 °C and 5% CO_2_. NHDFs were infected with clinical HCMV (either strain TR^31^ or TB40E-GFP^34^) at an MOI of 0.05 and allowed to proceed to 90% CPE. Each huBLT mouse was pre-treated with 1 mL of 4% Thioglycollate (Brewer’s Media, BD) by intraperitoneal (IP) injection to recruit monocyte/macrophages. At 24 hrs post-treatment, huBLT mice were infected with HCMV-infected fibroblasts from two T150 flasks (approximately 10^5^ PFU of cell-associated virus per mouse) via IP injection. Mock-infected huBLT mice were infected with uninfected fibroblasts from two T150 flasks.

### Treatment of huBLT mice with G-CSF for viral reactivation

To promote HCMV reactivation, mice were treated with G-CSF and AMD3100 as previously described^31^. Briefly, G-CSF (Neupogen, 100 uL at 300 ug/mL, Amgen) was delivered via an osmotic pump (1007D, Azlet) surgically implanted in the subcutaneous space beneath the dorsal skin on the mouse for 7 days. Additionally, on the same day as pump implantation, mice were also given a single IP injection of AMD3100 (1,1′-[1,4-Phenylenebis(methylene)]bis-1,4,8,11-tetraazacyclotetradecane octahydrochloride at 125 μg).

### Detection of HCMV genomes by qPCR

Total DNA was extracted from approximately 1 mm^2^ sections of mouse spleen or liver using the DNAzol reagent (Life Technologies). HCMV genomes were analyzed using quantitative PCR (TaqMan) performed on 1 ug of total DNA and using TaqMan FastAdvance PCR Master Mix (Applied Biosystems), according to the manufacturer’s instructions. Primers and a probe recognizing HCMV UL141 were used to quantify HCMV genomes (probe: CGAGGGAGAGCAAGTT; forward primer: 5′GATGTGGGCCGAGAATTATGA and reverse primer: 5′ATGGGCCAGGAGTGTGTCA). The probe contains a 5′ FAM reporter molecule and a 3′ quencher molecule (Applied Biosystems). The reaction was activated at 95 °C for 10 minutes followed by 40 cycles (15 s at 95 °C and 1 min at 60 °C) using a StepOnePlus TaqMan PCR machine. Results were analyzed using ABI StepOne software (Applied Biosystems).

### Generation of Autologous B-lymphoblastoid cell lines (B-LCLs)

Autologous fetal liver mononuclear cells (2 × 10^7^) were obtained from the flow-through (CD34-) fraction obtained after CD34+ HPC isolation. B-LCLs were derived by infecting mononuclear cells with EBV (B958) as previously described^38^ in RPMI (Cellgro) containing 20%FBS, 1% penicillin, 1% streptomycin, 1% glutamine and cyclosporine A. At 10 days post-infection, cells were maintained for an additional 6 weeks in RPMI containing 10%FBS, 1% penicillin, 1% streptomycin, 1% glutamine and 1% HEPES with twice-weekly media changes prior to expansion.

### HCMV-specific ELISPOT analysis

Human interferon gamma (IFNγ) enzyme linked immunospot assasys (ELISPOT, MabTech) were used to quantify HCMV-specific T-cell responses in leukocytes. Isolated mononuclear cells from HCMV-infected or uninfected huBLT mice were obtained from spleen sections either through density gradient separation using Ficoll (BD Bioscience) or isolation of human CD4 and CD8-specific subsets using magnetic bead separation (Miltenyi Biotech, purity greater than 80%). T-cell subsets or mononuclear cells were plated at 10^5^ cells per well in RPMI (Cellgro) containing 10% FBS (HyClone), and 1% penicillin, 1% streptomycin and 1% glutamine (Life Technologies). Unstimulated controls for each assay included either media alone or LCLs alone and for a positive control, samples were stimulated with *Stapylococcus aureus* enterotoxin B (SEB). Samples were incubated with either HCMV virion lysate or LCLs (10^5^ cells per well) pre-loaded with HCMV-specific peptides (pp65 or IE, JPT peptides). All samples were analyzed for human IFNγ using the Human IFNγ ELISPOT plates (MabTech), incubated for 56 hrs at 37 °C with 5% CO_2_ and developed according to the manufacturer’s instructions. Plates were imaged and spots counted using the AID ELISpot Reader.

### HCMV microneutralization assay

The microneutralization assay was adapted from a previously reported technique^50^. Briefly, human fibroblasts (MRC-5) were plated (2.0–2.5 × 10^4^/well) into flat-bottom 96-well plates in 100 μl of DMEM as described above and cultured for 24 hrs at 37 °C in 5% CO_2_. huBLT mouse serum was serially diluted two-fold and 50 uL of each dilution incubated with 200 plaque forming units of the clinical strain HCMV TR for 1 h at 37 °C in DMEM containing 5% FBS. The mixtures were added to the fibroblast monolayers and centrifuged at 2,000 g for 30 min prior to incubation at 37 °C for 90 min. Growth medium (100 μl) was added and the cultures incubated for an additional 96 hrs. The cells were fixed with 100% methanol for 10 min at −20 °C, rehydrated in PBS for 10 min, blocked with 5% FBS in PBS for 30 min, and then incubated with a mouse anti-CMV (0898, Santa-Cruz, sc-58118; 1:1000) antibody diluted in blocking buffer for 1 hr. The cells were then washed 3 times with PBS containing 0.05% Tween-20 and incubated with a goat anti-mouse IgG-HRP (Santa Cruz, sc-2005; 1:1000) secondary antibody diluted in blocking buffer for 1 hr, washed as above and a peroxidase substrate (TrueBlue, KPL) added for 8–15 min. Positivity was defined as ≥40% inhibition of HCMV positive cells, compared to negative control.

### Anti-HCMV ELISA

Antibody responses following HCMV infection of huBLT mice were assessed by enzyme-linked immunosorbent assay (ELISA). Costar clear polystyrene high-protein-binding enzyme immunoassay (EIA) plates (Corning) were coated in either purified HCMV virion preparations (5 ug/mL) diluted in PBS or recombinant CMV gB (0.75 ug/mL; Abcam, ab43040) diluted in a coating buffer composed of 0.015 M Na_2_CO_3_ and 0.035 M NaHCO_3_ (Corning). After overnight incubation, plates were washed five times with 200 μl of wash buffer (PBS containing 0.25% Tween 20) then blocked for 90 min at room temperature in blocking buffer (wash buffer containing 0.89% bovine serum albumin). huBLT mouse serum was serially diluted 2-fold in blocking buffer, plated at 100 μl per well and incubated at 37 °C for 45 min. Plates were washed as above and incubated with 50 μl of secondary antibody (either anti-IgA/M/G, #609-103-130; anti-IgG, #609-1312; or anti-IgM, #609-1307, all from Rockland Inc.) for 30 min at 37 °C. Plates were washed as above prior to visualization and quantification of antibody binding by addition of chromogen OPB substrate (Invitrogen). Optical densities at 450 nm were determined using an ELISA plate reader (Synergy HTX Multi-mode Reader, BioTex). Endpoint antibody titers were calculated using log-log transformation of the linear portion of the curve.

### Statistical Analysis

Statistical analysis was performed using GraphPad Prism (v6) for comparison between groups using one-way ANOVA. Values are expressed as mean+/− standard error of the mean. Significance was accepted with p < 0.05.
